# Human Genome Epidemiology: A Scientific Foundation for Using Genetic Information to Improve Health and Prevent Disease

**Published:** 2005-03-15

**Figure:**
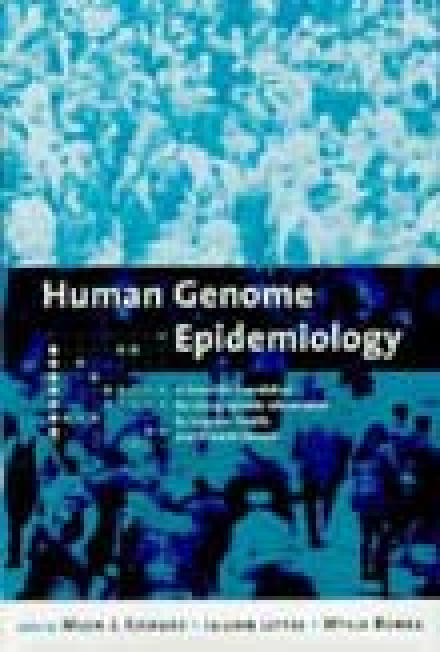


*Human Genome Epidemiology* is edited by three well-recognized experts in genetic epidemiology, and their experience shows in the information and organization of this text. While they do not define their intended audience, the material focuses on the complexities of epidemiologic study and design in population-based genomics. Readability characterizes all the chapters, and the concepts will be accessible to anyone with a firm grounding in epidemiologic principles. Hence, the book will be useful for both the graduate student and the experienced practitioner who wish to learn more about this field.

The book contains four parts. Part One is devoted to the basics of common definitions, molecular biology, and the laboratory testing that drove the Human Genome Project and continue to be central in genomic research. Definitions include distinguishing *genetics* (the study of single genes and their effects) from *genomics* (the study of both single genes and the functions and interactions of all genes in the genome). An example of the editors' thorough attention to detail is a chapter on the ethics related to genomic studies.

Part Two is the most technical aspect of the text and examines the application of standard epidemiologic research techniques in human genomics. These chapters discuss additional research methods unique to human genome epidemiology and provide comprehensive descriptions of the strengths and drawbacks of each research technique. The final chapter in this section provides a checklist for designing and reporting genomic epidemiologic studies. At this point, the average epidemiologist should have the information necessary for assessing existing studies and perhaps designing credible original studies as well.

How useful will the results of these studies prove to practical disease prevention? Part Three explores three types of validity for human genetic tests. *Analytic validity* is the sensitivity, specificity, and predictive value of a laboratory test for genotype. *Clinical validity* refers to the same values with respect to phenotype; that is, is a given test a good measure of gene–disease association?  Last, *clinical utility* refers to whether such tests provide information that can change disease outcomes. The remainder of this section discusses the application of these concepts to clinical medicine, pharmacology, and randomized clinical trials and offers guidelines for assessing these values in different settings.

Part Three leaves the reader with a sense that while genetic testing may be useful in clinical contexts or among select high-risk populations, there is little evidence to support the use of most genetic tests in mass population testing. Part Four reinforces this concept by providing 12 case studies on using human genome epidemiology to improve health. In one example after another, reasonable evidence often supports an association between a health outcome and a gene, a gene–gene interaction, or a gene–environment interaction. But never does the evidence suggest that general population testing would be an appropriate public health response.

One might infer that this text indicates a limited role for human genomic epidemiology in current public health practice. However, the book does offer important points for practical epidemiology. This is a rapidly growing field, and research epidemiologists can play an essential role in designing and conducting research. In fact, we may learn tomorrow of genetic tests with immediate general population applications. When such reports do become available, it will be essential for epidemiologists to quickly grasp the strengths and limitations of the interpreted studies. As genetic testing becomes a high-profile public discussion, epidemiologists will need to provide policy makers with a clear understanding of how to separate the wheat from the chaff in determining public health actions. The editors note that the results of genomic epidemiologic studies can be explored by other disciplines: policy (to decide value added), communication (to explain risk information), economics (to examine cost effectiveness), and outcomes research (to measure impact). For the most part, these aspects are not discussed in this text, but public health professionals will need to address them all to use human genomic epidemiology effectively in practice.

